# Fisk Prize Fund

**Published:** 1850-01

**Authors:** 


*Fisk Prize Fund.*—The trustees of this fund have selected for the year 1849–50, the two following subjects, and offer a premium of fifty dollars for the best dissertation on each, viz.:

1st. The History of Medical Delusions of the present and former times.

2d. Asiatic Cholera (so called). Its history, nature, and best mode of treatment.

Competitors are expected to transmit, free of expense, to one of the trustees, or the secretary of the board, on or before the first Wednesday in May, 1850, their dissertations with a motto written thereupon, and accompanied with a sealed letter having the same motto inscribed upon the outside, and their name and place of residence within. The award of the trustees will be made known at the annual meeting of the Rhode Island Medical Society, to be held at Providence, on the last Wednesday in June, 1850. L. Augustus Arnold, M. D., and George Capron, M. D., of Providence, and Hiram Allen, M. D., of Cumberland, R. I. *Trustees*, Thomas H. Webb, M. D., of Boston, *Secretary.*—*New York Journal of Medicine and Col. Sciences.*

